# Task-dependent learning of non-adjacent dependencies: Success in a familiarity rating task but failure in a two-alternative forced-choice task

**DOI:** 10.3758/s13423-025-02824-0

**Published:** 2025-12-15

**Authors:** Helen Shiyang Lu, Toben H. Mintz

**Affiliations:** 1 Department of Psychology, University of Southern California, Los Angeles, CA USA; 2 Present Address: School of Audiology and Speech Sciences, University of British Columbia, Vancouver, BC Canada; 3 Department of Psychology and Department of Linguistics, University of Southern California, Los Angeles, CA USA

**Keywords:** Non-adjacent dependency, Statistical learning, Ratings, 2AFC

## Abstract

Acquiring non-adjacent dependencies (NADs) from continuous sequences can be challenging for adults, with prior research showing varied outcomes depending on the properties of the stimuli and methods of assessments. This study investigated whether different behavioral tasks vary in their ability to detect NAD learning. All participants (*N* = 322) underwent equivalent training phases involving exposure to NAD trigrams in a continuous speech stream. During the test phase, their learning of the grammatical NAD patterns was evaluated using either a two-alternative forced-choice (2AFC, *N* = 200) task or a familiarity rating task (*N* = 122). Participants in the 2AFC task performed at chance, regardless of whether the ungrammatical trigram differed minimally or maximally from the grammatical trigram. In contrast, participants in the familiarity rating task rated grammatical trigrams as more familiar than ungrammatical ones, suggesting that the familiarity rating task may be more sensitive to subtle learning effects. These findings highlight the importance of task design in the detection of NAD learning, with implications for the broader field of statistical learning research.

**Table 1:** Examples of training, test trial, and catch trial trigrams

Training	Test trial		Catch trial	
	Grammatical	Ungrammatical	Novel	Part
$aX_{\{1,2,3\}}b$	$aX_{\{4,5,6,7,8,9\}}b$	$aX_{\{4,5,6\}}f$ $aX_{\{7,8,9\}}d$	*img* *jnk*	$X_1bc$ $faX_1$
$cX_{\{4,5,6\}}d$	$cX_{\{1,2,3,7,8,9\}}d$	$cX_{\{1,2,3\}}f$ $cX_{\{7,8,9\}}b$	*njh* *khl*	$X_4de$ $bcX_4$
$eX_{\{7,8,9\}}f$	$eX_{\{1,2,3,4,5,6\}}f$	$eX_{\{1,2,3\}}d$ $eX_{\{4,5,6\}}b$	*gli* *mik*	$X_7fa$ $deX_7$

Note. Here, each letter represents one artificial word. The assignment of actual artificial words to letters was randomized across participants

## Introduction

Non-adjacent dependencies (NADs) refer to the frequent co-occurrences of elements in a sequence that are separated by one or more intervening elements. For example, in the phrase *the cat that chased the mouse was hungry* the relationship between *cat* and *was* constitutes a non-adjacent dependency, with *that chased the mouse* serving as the intervening elements. NADs are common features of human language, allowing speakers to form complex and hierarchically structured sentences. Understanding how they are learned can provide useful insights into language acquisition and cognitive processing.

Prior research has extensively explored the factors influencing NAD learning, primarily focusing on the characteristics of the training stimuli. Acoustic cues such as pauses and prosody are helpful as they often signal boundaries within speech and can highlight important structural relationships between non-adjacent elements (Peña et al., 2002; Grama et al., 2016; Langus et al., 2012). Similarly, rhythmic and top-down structural cues facilitate the processing of NADs (Wang et al., 2019, 2017). Furthermore, increased variability in the interveners can enhance the abstraction of non-adjacent rules by decreasing the utility of learning simpler adjacent dependencies (Gómez, 2002). As learners become more adept at NAD learning, the need for high variability decreases (Gómez and Maye, 2005). The perceptual similarity between dependent items further facilitates NAD learning, as evidenced by studies showing that similar pitches or vowel properties aid in learning NADs for adults and infants (Newport and Aslin, 2004; Gebhart et al., 2009; Grama et al., 2016; Weyers et al., 2022).

This paper shifts the focus from the characteristics of the training stimuli to the methods used to assess adults’ NAD learning outcomes. The choice of task can influence the sensitivity of the measure and reliability of results. In this study, we compare two common behavioral tasks used in statistical learning research: the two-alternative forced-choice (2AFC) task and the familiarity rating task. Both tasks require participants to make explicit judgments about the test items, but they differ in response format. The 2AFC task requires participants to make a binary decision, demanding a clear and conscious judgment that may overshadow more subtle, intuitive knowledge. In contrast, the familiarity rating task allows for a more nuanced response, where participants rate their familiarity on a scale. This graded response format might be more sensitive to detecting subtle learning effects. Siegelman, Bogaerts, Christiansen, and Frost (2017) have pointed out the limitations of offline tasks like the 2AFC in accurately capturing learning dynamics, emphasizing that task design can greatly affect the detection of statistical learning and the interpretation of individual differences in learning ability.

A growing body of work suggests that the choice of test format can influence the extent to which statistical learning is detected, in part because different tasks may engage different forms of memory or knowledge (Batterink et al., 2015; Liu et al., 2023; Isbilen et al., 2020; Kidd et al., 2020). For example, Liu et al. (2023) dissociated implicit and explicit traces by showing that they differ in both forgetting rates and susceptibility to testing effects. Furthermore, performance on direct and indirect tasks was uncorrelated across individuals, supporting the idea that these tasks tap into distinct memory systems. Similarly, Batterink et al. (2015) found that a forced-choice recognition task and a reaction-time-based detection task captured distinct aspects of learning: the former primarily reflected explicit knowledge, while the latter revealed implicit learning even in participants who could not consciously recognize the learned items. Performance across the two tasks was again uncorrelated. However, much of this evidence comes from studies of adjacent transitional probabilities. It remains less clear how task format affects learning outcomes for more complex structures, such as NADs, which are known to place heavier demands on attention and working memory (Conway, 2020). Prior work by Wang et al. (2019) has raised the possibility that familiarity rating tasks may be more sensitive to NAD learning than forced-choice formats, but direct comparisons remain rare. The present study aims to address this gap by directly comparing 2AFC and familiarity rating tasks in a challenging NAD learning context. By comparing these two tasks, this study aims to shed light on how task design influences the detection of NAD learning and contribute to the optimization of methodologies for future research in statistical learning.

## Methods

### Stimuli

A female speaker recorded fifteen monosyllabic artificial words – *bep, bok, des, dob, feep, foom, foop, ghan, gop, ghep, kag, meb, mak, pag, poog* – digitized at 44.1kHz. Using the *lengthen* function in Praat (Boersma, 2001), these recordings were modified to have a uniform duration of 250 ms each. To improve clarity, an additional 83 ms of silence was appended to the end of each word, bringing their total length to 333 ms. Research has indicated that this speech rate facilitates the learning of NADs in adults when no other cues are present (Wang et al., 2019).

Three NADs were constructed using six of the artificial words: $a\_b$, $c\_d$, and $e\_f$, where the letters indicate unique words. Nine other words were placed between these pairs to create nine distinct NAD trigrams: $aX_{\{1,2,3\}}b$, $cX_{\{4,5,6\}}d$, and $eX_{\{7,8,9\}}f$. The assignment of specific words to these letters was randomized for each participant to ensure that the results were not influenced by the particular word combinations used in the NADs.

For the testing phase, 18 new grammatical trigrams and 18 new ungrammatical trigrams were generated. The grammatical trigrams combined a previously learned NAD with a middle element that had not been used with that NAD during training, resulting in 18 unique combinations (refer to Table 1). Each ungrammatical trigram was created by altering the final word of a grammatical trigram in a way that broke the learned NAD, but also ensuring that the middle word had not appeared with either the first or last word during training (e.g., $aX_{4}f$, where $a\_f$ disrupted the NAD and $X_4$ was never paired with either *a* or *f*). This manipulation maintained adjacent transitional probabilities at zero for both grammatical and ungrammatical trigrams. In addition, all words in the test trigrams were familiar to participants from the training phase and occurred in their attested trigram positions. This way, the only way to differentiate between the two types of trigrams was by learning the NADs.

Since this study was conducted online, we created novel and part-trigrams to ensure that participants were paying attention to the task. An additional eight monosyllabic artificial words were created for generating the novel trigrams (i.e., *fod, ghen, goob, keb, koog, mep, seeg, soog*). Novel trigrams were formed by randomly combining three of the eight artificial words. If participants were paying attention, they should find these unfamiliar.

The part-trigrams were created by taking three consecutive words that crossed trigram boundaries during training (e.g., for words $aX_{\{1,2,3\}}b$, examples of part words could be $X_1bc$ and $faX_1$). If participants were paying attention to the task, they would find the part-trigrams more familiar than the novel trigrams.

### Procedure

Participants were instructed to complete the experiment in a quiet environment on their computer. They were informed that they would be listening to a sequence of novel words and that they would later be asked questions about what they heard.

Participants underwent three blocks of training and testing phases. For the testing phases, participants were randomly assigned to one of the three tasks: 2AFC minimal difference (where the ungrammatical and grammatical trigram within a pair only differed by one word), 2AFC maximal difference (where the ungrammatical and grammatical trigram within a pair differed by all three words), or familiarity rating. The training phases remained structurally equivalent across the three tasks. Each participant only experienced one of the three tasks throughout the experiment.

#### Training phase

Building on the methodology used in Wang et al. ’s (2019) study, each training phase included 16 repetitions of the nine trigrams, for a total of 144 NAD trigrams. The trigrams were pseudo-randomly combined into a continuous speech stream, ensuring that no adjacent trigrams featured the same NAD. The 144-second speech stream contained no pauses, prosodic cues, or other acoustic markers to indicate the boundaries between NAD trigrams, so that participants had to rely on distributional information to learn the dependencies. During training, participants were simply required to listen passively to the speech stream, and their computer had a blank screen displayed.

Across the three blocks, the training phases followed the same pseudo-random structure, with the same number of repetitions of each trigram and the same constraints on ordering, but the exact sequence of trigrams varied across blocks due to randomization.

#### Testing phase

A testing phase started after a training phase concluded. In each of the three testing phases, participants were presented with 12 test trials and 6 catch trials. Across the three blocks, all 36 possible test trigrams (18 grammatical and 18 ungrammatical) were tested. In the 2AFC task, the 18 grammatical and 18 ungrammatical trigrams were randomly paired to form 18 unique pairs. Each block included 6 unique pairs, and each pair was presented twice (once with the grammatical trigram first, once with the ungrammatical first), resulting in 12 test trials per block. This ensured that any potential order effects were counterbalanced. In the familiarity rating task, each block included 6 unique grammatical and 6 unique ungrammatical test trigrams, such that across the three blocks, participants rated all test items once. The use of multiple blocks allowed us to examine cumulative learning effects and potential changes in performance across time.

##### 2AFC task

The test trials presented one grammatical and one ungrammatical trigram within a pair, and the catch trials presented one novel and one part trigram within a pair. Each test pair was presented twice: once with the grammatical or part- trigram first and once with the ungrammatical or novel trigram first. For the catch trials, six novel trigrams and six part-trigrams were newly generated in each block, so that participants would not encounter the same novel or part-trigram across different blocks.

**Table 2:** Summary of participants’ age and gender information in each condition

Condition	Age (years)				Gender			Sample size	
	Mean	SD	Min	Max	Female	Male	Non-binary	Excluded	Included
2AFC minimal	20.08	1.09	18	23	71	25	0	6	96
2AFC maximal	19.81	1.08	17	23	62	30	4	2	96
Familiarity rating	19.93	1.16	17	22	61	36	0	25	97

*Note*. The age and gender information is based on the included participants only.

Each trial presented two trigrams sequentially, separated by a one-second silence. When the two trigrams were presented, participants saw on screen "First sequence" and "Second sequence" to help them better differentiate between the two. After hearing the two trigrams, participants were prompted to select whether the first or second sequence sounded more familiar to them. The order of trials was pseudo-random for each participant, such that no more than two trials had the same type of sequences presented first within a pair (grammatical, ungrammatical, novel, or part). Once participants made their selection, the next trial started.

##### Minimal difference

In the minimal difference condition, the two trigrams within a test trial only differed by one word. For example, if the grammatical trigram was $aX_{4}b$, the paired ungrammatical trigram could be $aX_{4}f$ or $eX_{4}b$.

##### Maximal difference

In the maximal difference condition, the two trigrams within a test trial shared no words in common. For example, if the grammatical trigram was $aX_{4}b$, the paired ungrammatical trigram could be any one in $cX_{\{1,2,3\}}f$ and $eX_{\{1,2,3\}}d$.

##### Familiarity rating task

The test trials presented either a grammatical or an ungrammatical trigram, and the catch trials presented a novel trigram. Part-trigrams were not included in the familiarity rating task. A new set of six novel trigrams was generated for each testing phase, so that participants could not encounter the same novel trigram across different blocks.

In a test trial, participants heard the same trigram twice, separated by a one-second silence. When the trigram was presented, participants saw on screen "Playing sequence" or "Repeating sequence" to help indicate that the same trigram was being played twice. After listening to the trigram twice, participants were presented with a sliding scale and the question, "Do you think that you heard this sequence in the previous section?" They were given five options to select from: "Definitely yes," "Maybe yes," "Not sure," "Maybe not," and "Definitely not." The next trial started after participants made their selection.

#### Exclusion criteria

For the catch trials in the 2AFC task, if participants paid attention to the training phase, they should have found the part-trigrams more familiar than the novel trigrams. If participants failed to select the part-trigram as more familiar than the novel trigram in at least four out of the six catch trials in a given block, then their data for that block was excluded from the analysis.

For the familiarity rating task, participants should have found the novel trigrams in catch trials unfamiliar. If participants rated the novel trigrams as "Not sure," "Maybe yes," or "Definitely yes" in more than two of the six catch trials in a given block, then their data for that block was excluded from the analysis.

For both tasks, all participants who passed the attention criterion for at least one block were included in the analysis, excluding only test blocks in which they failed the attention criterion.

#### Transparency and openness

We report all data exclusions and manipulations in the study. Sample research materials, all raw data, and the analysis scripts are available at https://osf.io/8wu5j. Data were analyzed using R, version 4.3.2 (R Core Team, 2021), the package *ordinal*, version 2022.11-16 (Christensen, 2019), the package *lme4*, version 1.1-34 (Bates et al., 2015), the package *brms*, version 2.20.3 (Bürkner, 2018), and the package *bayestestR*, version 0.13.1 (Makowski et al., 2019). In the data analysis report on our OSF repository, we also report the results of all ordinal logistic regression models, binomial generalized linear mixed models, Bayesian regression models, and their tests for practical equivalence.

### Participants

A total of 322 participants were recruited from the the university’s Psychology Subject Database. The study’s protocol was approved by the university’s institutional review board, and participants gave informed consent at the beginning of the session. Participants received course credits for their time in the study. After excluding participants who failed the attention check in all three blocks, the final sample consisted of 289 participants: 96 in the 2AFC minimal difference condition, 96 in the 2AFC maximal difference condition, and 97 in the familiarity rating condition. See Table 2 for a breakdown of participants’ age and gender in each condition.

## Results

We fitted mixed-effects models appropriate to each task type (binomial generalized linear mixed models for 2AFC; ordinal logistic regressions for familiarity rating), using the *glmer* function in the *lme4* package (Bates et al., 2015) and the *clmm* function in the *ordinal* package (Christensen, 2019). Because of differences in task design and outcome measures, we selected the best fitting model within each task type to appropriately capture effects in each dataset. The best-fitting model was selected based on the Akaike Information Criterion, and reported in this paper. All fitted models and their results are available in the analysis script on the OSF repository. Across models, the conclusions regarding learning success versus failure did not change: 2AFC performance remained at chance, and familiarity ratings showed a numerical preference for grammatical trigrams. In a few alternative models for the rating task, the grammaticality effect was marginal ($p <.06$), but the direction of the effect was consistent.

**Table 3:** Summary of participants’ accuracy in the 2AFC tasks for each block

Block	Minimal difference			Maximal difference		
	Mean	SD	n	Mean	SD	n
1	0.52	0.15	84	0.52	0.13	85
2	0.48	0.15	63	0.52	0.13	76
3	0.52	0.15	71	0.51	0.13	70
All	0.51	0.10	96	0.52	0.09	96

*Note.* Sample sizes (n) vary across blocks due to participants failing attention checks in specific blocks, resulting in the removal of data for that block only

**Fig. 1:**
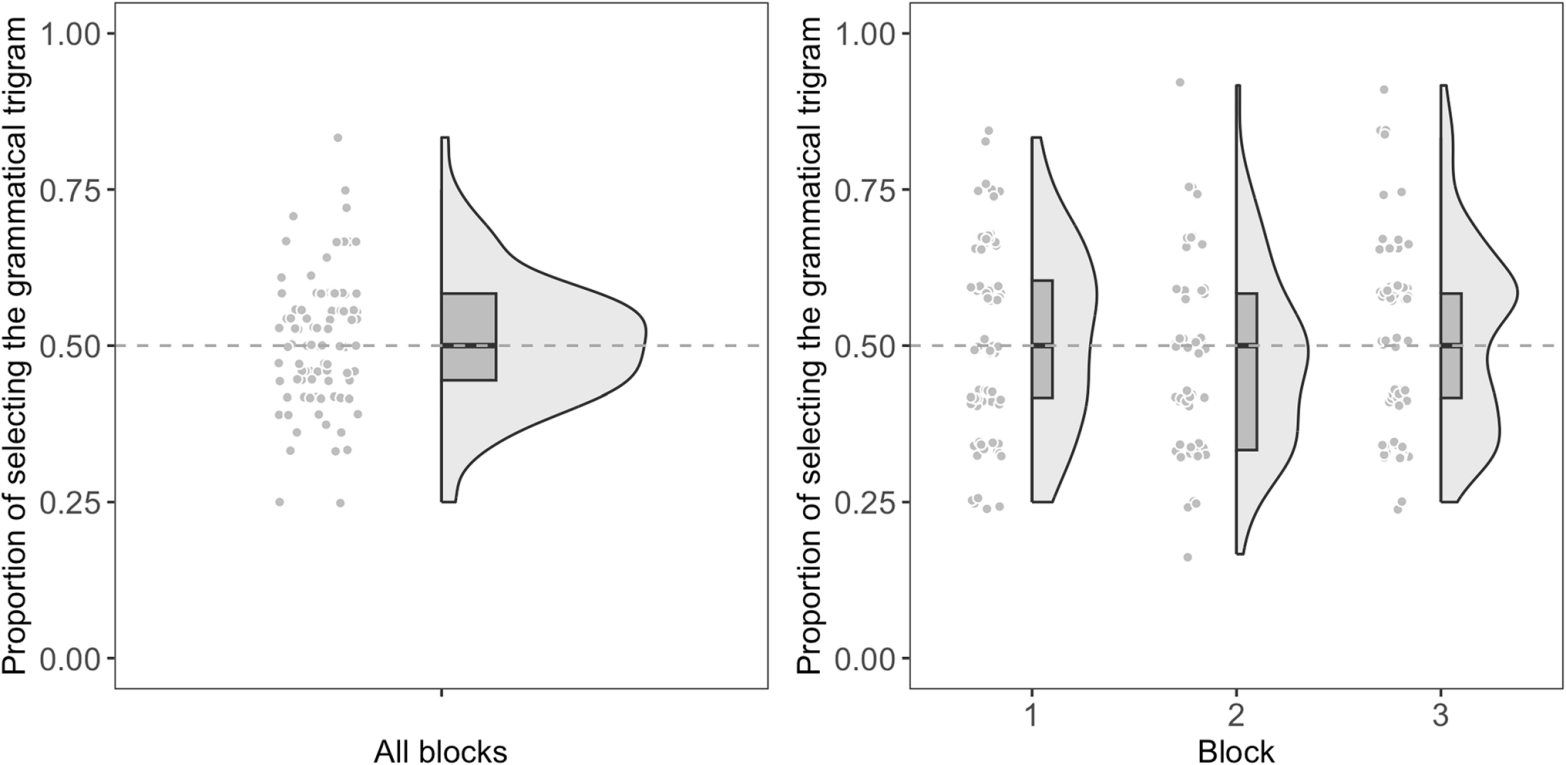
Each participant’s accuracy in the 2AFC task for the minimal difference condition. The *left panel* shows the accuracy for all blocks combined, and the *right panel* shows the accuracy for each block

### 2AFC task

We coded participants’ selection of grammatical trigram as 1 (i.e., correct) and ungrammatical trigram as 0 (i.e., incorrect). We then calculated participants’ accuracy for each block as well as for all blocks combined. If participants were able to learn the NADs during the training phase, they should have an accuracy above the chance level of 0.50 in the 2AFC task.

Fo each condition (i.e., minimal difference and maximal difference), we fitted all possible binomial generalized linear mixed models, including the main effects of the first trigram type (i.e., whether the grammatical or ungrammatical trigram was presented first, sum-coded), block (1 vs. 2 vs. 3), test trial number within a block, and their possible interactions, as well as by-subject random effects. In these models, an intercept of 0 corresponds to log-odds of 0, which maps to a response probability of 0.50 (i.e., chance performance). If participants were able to learn the NADs, we should see that the intercept of the model differs significantly from 0.

**Fig. 2:**
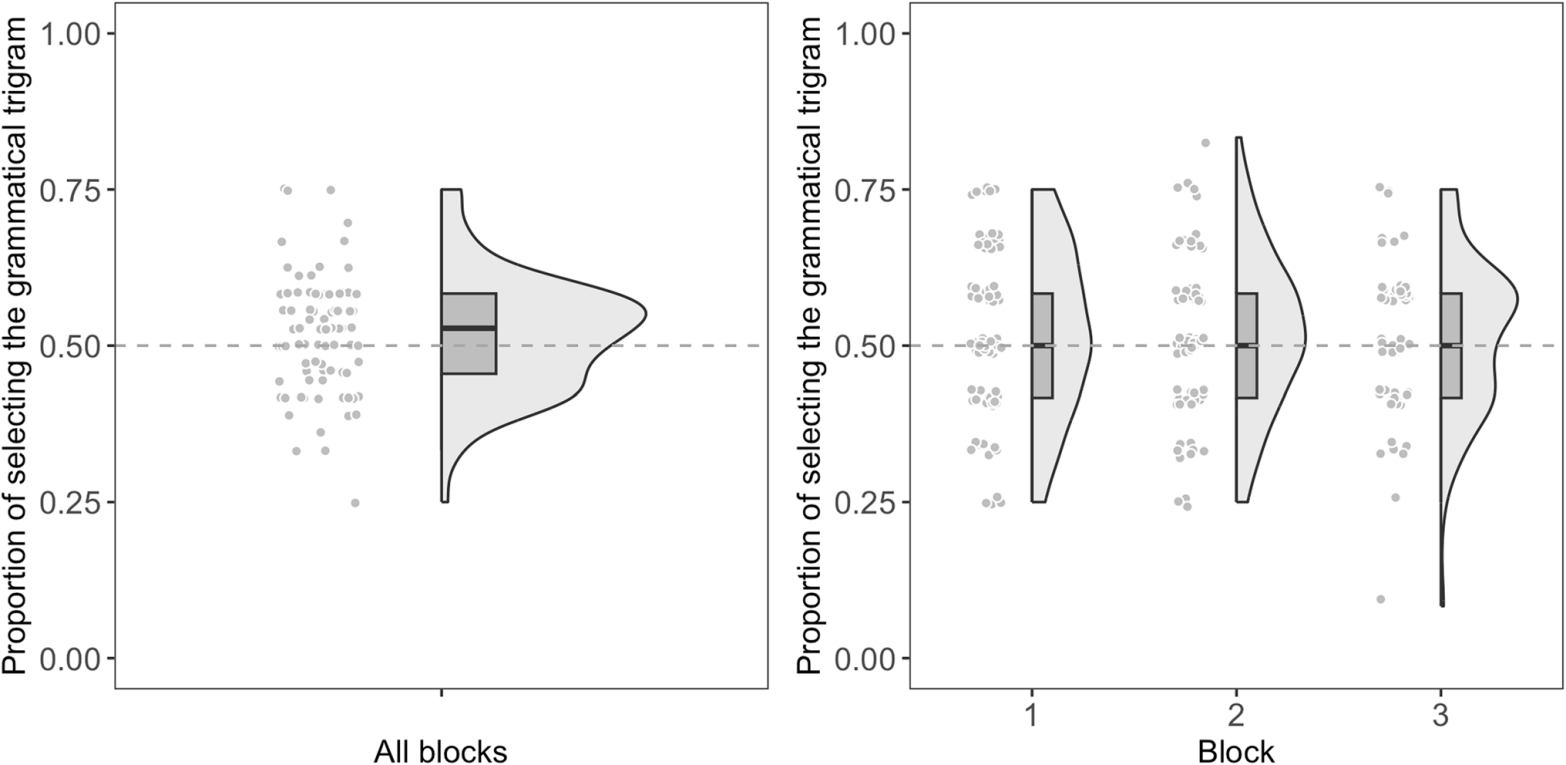
Each participant’s accuracy in the 2AFC task for the maximal difference condition. The *left panel* shows the accuracy for all blocks combined, and the *right panel* shows the accuracy for each block

#### Minimal difference

Participants’ accuracy across the test phase is shown in Table 3. The best-fitting model included the main effects of the first trigram type, block, and their interaction, as well as a by-subject random intercept. The model did not find the intercept to be significantly different from 0 ($\beta$ = 0.03, *SE* = 0.10, *z* = 0.29, *p* = .773, *OR*: 1.03, $95\% CI$ [0.83, 1.23]). It also did not find the effect of the block to be significant ($\beta$ = -0.003, *SE* = 0.05, *z* = -0.06, *p* = .954, *OR*: 1.00, $95\% CI$ [0.91, 1.09]). On the other hand, the model found the effect of first trigram type ($\beta$ = 0.59, *SE* = 0.10, *z* = 5.85, *p* < .001, *OR*: 1.80, $95\% CI$ [1.60, 1.99]) and its interaction with block ($\beta$ = -0.19, *SE* = 0.05, *z* = -3.99, *p* < .001, *OR*: 0.83, $95\% CI$ [0.74, 0.92]) to be significant. Participants were more likely to answer the question correctly when the grammatical trigram was presented first, and this effect faded as they experienced more blocks. Figure 1 shows each participant’s accuracy in the 2AFC task for the minimal difference condition.

Since we are mostly interested in interpreting the model’s intercept (i.e., whether participants were able to learn the NADs during the training phase), we also fitted a base model that only included a by-subject random intercept. The model again did not find the intercept to be significantly different from 0 ($\beta$ = 0.02, *SE* = 0.04, *z* = 0.59, *p* = .558, *OR*: 1.02, $95\% CI$ [0.95, 1.11]).

We also fitted the same base model using a Bayesian logistic regression, with the *brm* function in the *brms* package, and performed a test for practical equivalence using the *equivalence_test* function in the *bayestestR* package. This helps us determine whether we can confidently claim that participants’ accuracy was equivalent to chance level (i.e., accept the null hypothesis). If the 89% highest density interval (HDI) falls within the pre-specified region of practical equivalence (ROPE), then we can accept the null hypothesis. The model found the 89% HDI of the intercept to be [-0.04, 0.09], which fell completely within the default ROPE of logistic regression models (i.e., [-0.18, 0.18]). Therefore, we can confidently claim that participants’ accuracy in the 2AFC minimal difference condition was equivalent to chance level.

#### Maximal difference

Participants’ accuracy across the test phase is again shown in Table 3. The best-fitting model included the main effects of the first trigram type and a by-subject random slope for the first trigram type. No other effects were included in the model. The model did not find the intercept to be significantly different from 0 ($\beta$ = 0.07, *SE* = 0.04, *z* = 1.84, *p* = .067, *OR*: 1.07, $95\% CI$ [1.00, 1.15]). It also did not find the effect of the first trigram type to be significant ($\beta$ = -0.04, *SE* = 0.05, *z* = -0.89, *p* = .373, *OR*: 0.96, $95\% CI$ [0.87, 1.05]). Figure 2 shows each participant’s accuracy for the maximal difference condition.

Since we did not find a significant effect of the first trigram type and we are mostly interested in interpreting the model’s intercept, we also fitted a base model that only included a by-subject random intercept. The model again did not find the intercept to significantly differ from 0 ($\beta$ = 0.07, *SE* = 0.04, *z* = 1.82, *p* = .068, *OR*: 1.07, $95\% CI$ [0.99, 1.15]).

Again, we fitted the same base model using a Bayesian logistic regression and performed a test for practical equivalence. The model found that the 89% HDI of the intercept falls completely within ROPE. Therefore, we can claim that participants’ accuracy in the 2AFC maximal difference condition was equivalent to chance level as well.

#### Comparison between minimal and maximal difference

We also compared participants’ accuracy between the minimal and maximal difference conditions. We fitted a binomial generalized linear mixed model to compare participants’ accuracy between the two conditions. The model included the main effects of condition (minimal vs. maximal, sum-coded) and by-subject random intercepts. We did not find the effect of condition to be significant ($\beta$ = 0.02, *SE* = 0.03, *z* = 0.85, *p* = .395, *OR*: 1.05, $95\% CI$ [0.97, 1.08]), indicating that participants’ accuracy did not differ between the two conditions. In addition, the model did not find the intercept to be significantly different from 0 ($\beta$ = 0.05, *SE* = 0.03, *z* = 1.69, *p* = .091, *OR*: 1.02, $95\% CI$ [0.99, 1.10]).

**Table 4:** Summary of participants’ ratings for the grammatical and ungrammatical trigrams for each block in the familiarity rating task

Block	Grammatical		Ungrammatical		n
	Mean	SD	Mean	SD	
1	3.83	0.52	3.63	0.58	81
2	3.75	0.56	3.54	0.54	58
3	3.55	0.70	3.40	0.66	63
All	3.73	0.53	3.53	0.53	97

*Note.* Sample sizes (n) vary across blocks due to participants failing attention checks in specific blocks, resulting in the removal of data for that block only

**Fig. 3:**
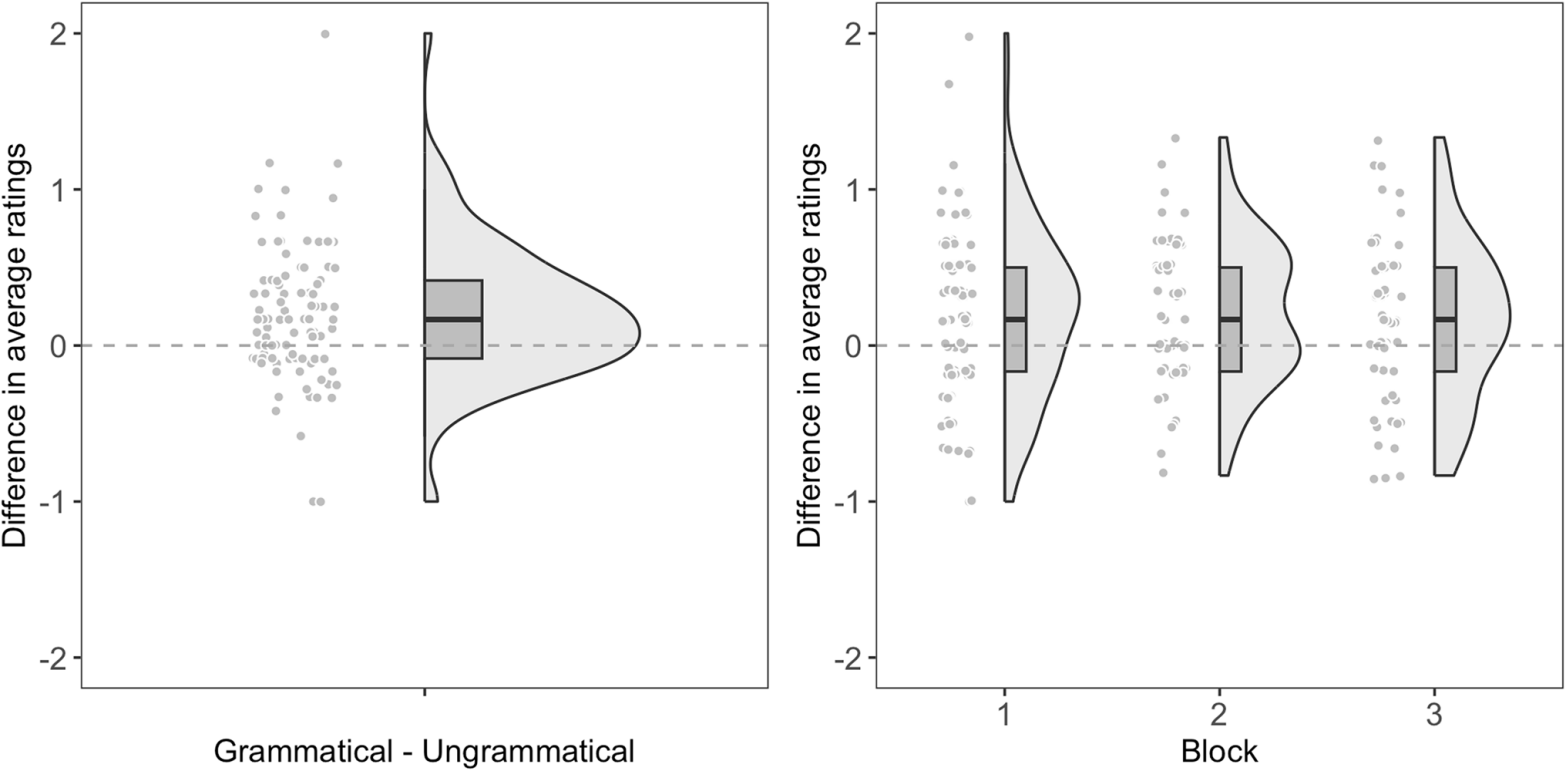
Each participant’s difference score in the familiarity rating task. The *left panel* shows the difference score for all blocks combined, and the *right panel* shows the difference score for each block

A Bayesian logistic regression model was also fitted, and the test for practical equivalence was performed. The model found that the 89% HDI of the intercept and that of the slope for condition both fall completely within ROPE. Therefore, we can claim that participants’ accuracy in the 2AFC minimal and maximal difference conditions were equivalent to chance level and did not differ depending on how the test pair was constructed.

### Familiarity rating task

We first converted participants’ ratings into an ordinal format (i.e., 5 = Definitely yes, 4 = Maybe yes, 3 = Not sure, 2 = Maybe not, 1 = Definitely not). We then calculated participants’ average ratings for the grammatical and ungrammatical trigrams for each block as well as for all blocks combined. If participants were able to learn the NADs during the training phase, they should have rated the grammatical trigrams as more familiar than the ungrammatical trigrams. Their average ratings are shown in Table 4 and their difference scores are shown in Fig. 3.

Ordinal logistic regression models were fitted to compare participants’ ratings for the grammatical and ungrammatical trigrams. We fitted all possible models, including the main effects of grammaticality (grammatical vs. ungrammatical, sum-coded), block (1 vs. 2 vs. 3), test trial number within a block (1 to 12), and their possible interactions, as well as by-subject random effects. If participants were able to learn the NADs during the training phase, we should see a significant effect of grammaticality, with participants rating the grammatical trigrams as more familiar than the ungrammatical trigrams.

The best-fitting model included the main effects of grammaticality, block, and test trial number, as well as by-subject random slopes for block. No interactions were included in the model. The model showed that participants were more likely to rate the grammatical trigrams as more familiar than the ungrammatical trigrams ($\beta$ = 0.15, *SE* = 0.04, *z* = 4.06, *p* < .001, *OR*: 1.17, $95\% CI$ [1.08, 1.25]). Participants’ ratings for the test trigrams dropped as trial number increased within a block ($\beta$ = -0.16, *SE* = 0.02, *z* = -7.21, *p* < .001, *OR*: 0.85, $95\% CI$ [0.82, 0.89]), and decreased as they experienced more blocks ($\beta$ = -0.15, *SE* = 0.06, *z* = -2.60, *p* = .009, *OR*: 0.86, $95\% CI$ [0.77, 0.96]).

## Discussion

This study examined non-adjacent dependency learning using two distinct behavioral tasks: a two-alternative forced-choice task and a familiarity rating task. The results revealed that while the familiarity rating task was successful in detecting evidence of NAD learning (replicating the findings of Wang et al. , 2019) the 2AFC tasks, regardless of how the testing pairs were constructed (i.e., with minimal or maximal similarity between the grammatical and ungrammatical trigrams), did not demonstrate learning above chance level. Additional Bayesian analyses suggested that participants indeed failed to learn NADs in the 2AFC tasks and that the null results were not due to a lack of statistical power. These findings altogether suggest that the nature of the task itself may significantly influence the detection of NAD learning. Therefore, it is crucial to consider the task’s design when comparing results across studies that investigate NAD learning.

The familiarity rating task’s success in indicating NAD learning suggests that tasks allowing for graded judgments may be more sensitive to subtle learning effects than binary forced-choice formats. While both tasks required explicit judgments, the graded response format may have allowed participants to express varying levels of familiarity, reducing decision noise and better capturing weak or uncertain knowledge. This interpretation is consistent with past research showing that task design and response format can strongly influence the detection of statistical learning effects (e.g., Kim et al. , 2009, Liu et al., 2023).

In contrast, neither the minimal nor maximal difference 2AFC tasks showed significant learning, with participant performance hovering around the chance level. While 2AFC tasks have been used successfully in studies of adjacent transitional probability word segmentation (e.g., Saffran et al. , 1996), they may not be well-suited for capturing the complexities of NAD learning. Additionally, we found no significant difference between the minimal and maximal conditions. Because performance in both conditions was at a chance level, it is difficult to draw strong conclusions about the potential role of similarity or dissimilarity in supporting recognition of NADs in this context. Nevertheless, we note that under these conditions, varying the similarity between test pairs failed to result in clear differences in performance.

We speculate that the following differences may have contributed to the divergent outcomes of the two tasks. To make the number of test trials equivalent, participants in the rating task heard each test trigram once, while participants in the 2AFC task heard each test pair twice, meaning that they heard repetitions of the same ungrammatical items. To test whether this repetition influenced performance, we conducted additional analyses that included repetition order as a predictor in the 2AFC models, and separate analyses that included only the first presentation of each test pair. In both cases, the results remained consistent: there was no evidence of learning, with performance remaining at chance. These results are reported in the OSF repository as additional analyses at https://osf.io/8wu5j/files/osfstorage/685a03a8581225f8c2d1648d. Thus, while prior work has suggested that reducing repetitions may improve test sensitivity and reliability (Siegelman, Bogaerts, & Frost, 2017), in this case, repetition alone did not appear to account for the difference in performance between the familiarity rating and 2AFC tasks. Therefore, other aspects of task design likely contributed to the differential sensitivity of these measures.

The two tasks may differ in their sensitivity to NAD learning due to their response formats. The 2AFC task places the dependent variable on a two-point scale, which may limit the granularity of the data and obscure subtle learning effects. Moreover, all test trigrams were novel combinations not encountered during training, intentionally designed to prevent chunking of familiar stimuli and to require generalization of the underlying NADs. However, this novelty also made the 2AFC task particularly challenging, as participants had to judge which of two unfamiliar trigrams was “more familiar”. In contrast, the familiarity rating task used a five-point scale and required only rating a single sequence’s familiarity, reducing cognitive load and allowing a more nuanced measure of knowledge. The graded responses captured a continuum of familiarity, potentially better reflecting varying degrees of learning.

Notably, prior studies have demonstrated NAD learning with 2AFC tasks, but typically under conditions with additional facilitating cues: such as perceptual similarity between dependent items (e.g., Newport and Aslin , 2004; Grama et al. , 2016), segmented streams with dependencies in prominent edge positions (e.g., Peña et al. , 2002; Wang and Mintz, 2018), or positional cues during testing (Frost and Monaghan, 2016). In contrast, our study used continuous streams with no prosodic or positional cues, low intervening variability, and novel test sequences. Under these more difficult conditions, 2AFC performance remained at chance, while the rating task revealed learning. These findings suggest that the success of 2AFC in detecting NAD learning may depend critically on supporting cues. Future work should examine how training and testing characteristics interact with task format to influence sensitivity to NAD learning.

In conclusion, this study highlights the importance of task selection in assessing NAD learning. While prior work has suggested that task format can influence sensitivity to statistical learning (e.g., Siegelman, Bogaerts, & Frost , 2017; Batterink et al., 2015; Liu et al., 2023), most direct comparisons of tasks have focused on simpler regularities such as adjacent dependencies. Here, we extend this work by showing that in a relatively challenging NAD learning paradigm without facilitating perceptual or positional cues, 2AFC tasks did not detect learning, whereas a familiarity rating task did. This finding suggests that graded rating tasks may be better suited for measuring NAD learning under these conditions. Our results suggest that the interaction between task format and target structure is critical: tasks that are effective for adjacent dependencies may fail for more complex patterns like NADs, especially when no supporting cues are present. These findings have important implications for the design and interpretation of statistical learning studies, highlighting the need to carefully match assessment methods to the nature of the learning task at hand.

## Data Availability

All data and sample materials have been made publicly available at the OSF Repository for this project at https://osf.io/8wu5j.

## Declarations

### Code Availability

The codes used for data analyses can be accessed at the project’s OSF repository https://osf.io/8wu5j.

## References

[CR1] Bates, D., Mächler, M., Bolker, B., & Walker, S. (2015). Fitting linear mixed-effects models using lme4. *Journal of Statistical Software, 67*(1), 1–48. 10.18637/jss.v067.i01

[CR2] Batterink, L. J., Reber, P. J., Neville, H. J., & Paller, K. A. (2015). Implicit and explicit contributions to statistical learning. *Journal of Memory and Language,**83*, 62–78. 26034344 10.1016/j.jml.2015.04.004 PMC4448134

[CR3] Boersma, P. (2001). Praat, a system for doing phonetics by computer. *Glot. Int.,**5*(9), 341–345.

[CR4] Bürkner, P.-C. (2018). Advanced Bayesian multilevel modeling with the R package brms. *The R Journal*, *10*(1), 395–411. 10.32614/RJ-2018-017

[CR5] Christensen, R. H. B. (2019). Ordinal—regression models for ordinal data. R package version 2019.12-10. https://CRAN.R-project.org/package=ordinal

[CR6] Conway, C. M. (2020). How does the brain learn environmental structure? Ten core principles for understanding the neurocognitive mechanisms of statistical learning. *Neuroscience & Biobehavioral Reviews,**112*, 279–299. 32018038 10.1016/j.neubiorev.2020.01.032 PMC7211144

[CR7] Frost, R. L., & Monaghan, P. (2016). Simultaneous segmentation and generalisation of non-adjacent dependencies from continuous speech. *Cognition,**147*, 70–74. 26638049 10.1016/j.cognition.2015.11.010

[CR8] Gebhart, A. L., Newport, E. L., & Aslin, R. N. (2009). Statistical learning of adjacent and nonadjacent dependencies among nonlinguistic sounds. *Psychonomic Bulletin & Review,**16*(3), 486–490. 10.3758/PBR.16.3.486 PMC2799498 19451373

[CR9] Gómez, R. L. (2002). Variability and detection of invariant structure. *Psychological Science,**13*(5), 431–436. 12219809 10.1111/1467-9280.00476

[CR10] Gómez, R. L., & Maye, J. (2005). The developmental trajectory of nonadjacent dependency learning. *Infancy,**7*(2), 183–206. 33430549 10.1207/s15327078in0702_4

[CR11] Grama, I. C., Kerkhoff, A., & Wijnen, F. (2016). Gleaning structure from sound: The role of prosodic contrast in learning non-adjacent dependencies. *Journal of Psycholinguistic Research,**45*(6), 1427–1449. 26861215 10.1007/s10936-016-9412-8 PMC5093218

[CR12] Isbilen, E. S., McCauley, S. M., Kidd, E., & Christiansen, M. H. (2020). Statistically induced chunking recall: A memory-based approach to statistical learning. *Cognitive Science,**44*(7), Article e12848. 10.1111/cogs.12848 32608077

[CR13] Kidd, E., Arciuli, J., Christiansen, M. H., Isbilen, E. S., Revius, K., & Smithson, M. (2020). Measuring children’s auditory statistical learning via serial recall. *Journal of Experimental Child Psychology,**200*, Article 104964. 32858420 10.1016/j.jecp.2020.104964

[CR14] Kim, R., Seitz, A., Feenstra, H., & Shams, L. (2009). Testing assumptions of statistical learning: Is it long-term and implicit? *Neuroscience Letters,**461*(2), 145–149. 19539701 10.1016/j.neulet.2009.06.030

[CR15] Langus, A., Marchetto, E., Bion, R. A. H., & Nespor, M. (2012). Can prosody be used to discover hierarchical structure in continuous speech? *Journal of Memory and Language,**66*(1), 285–306.

[CR16] Liu, H., Forest, T. A., Duncan, K., & Finn, A. S. (2023). What sticks after statistical learning: The persistence of implicit versus explicit memory traces. *Cognition,**236*, Article 105439. 36934685 10.1016/j.cognition.2023.105439

[CR17] Makowski, D., Ben-Shachar, M. S., & Lüdecke, D. (2019). Bayestestr: Describing effects and their uncertainty, existence and significance within the bayesian framework. *Journal of Open Source Software, 4*(40), 1541. 10.21105/joss.01541

[CR18] Newport, E. L., & Aslin, R. N. (2004). Learning at a distance I. Statistical learning of non-adjacent dependencies. *Cognitive Psychology, 48*(2), 127–162. 10.1016/s0010-0285(03)00128-2 14732409

[CR19] Peña, M., Bonatti, L. L., Nespor, M., & Mehler, J. (2002). Signal-driven computations in speech processing. *Science,**298*(5593), 604–607. 12202684 10.1126/science.1072901

[CR20] R Core Team. (2021). R: A language and environment for statistical computing. R Foundation for Statistical Computing. Vienna, Austria. Retrieved from https://www.R-project.org/

[CR21] Saffran, J. R., Newport, E. L., & Aslin, R. N. (1996). Word segmentation: The role of distributional cues. *Journal of Memory and Language,**35*(4), 606–621.

[CR22] Siegelman, N., Bogaerts, L., Christiansen, M. H., & Frost, R. (2017). Towards a theory of individual differences in statistical learning. *Philosophical Transactions of the Royal Society B: Biological Sciences,**372*(1711), 20160059. 10.1098/rstb.2016.0059 PMC5124084 27872377

[CR23] Siegelman, N., Bogaerts, L., & Frost, R. (2017). Measuring individual differences in statistical learning: Current pitfalls and possible solutions. *Behavior Research Methods,**49*, 418–432. 10.3758/s13428-016-0719-z PMC5011036 26944577

[CR24] Wang, F. H., & Mintz, T. H. (2018). Learning nonadjacent dependencies embedded in sentences of an artificial language: When learning breaks down. *Journal of Experimental Psychology: Learning, Memory, and Cognition,**44*(4), 604–614. 29608079 10.1037/xlm0000483

[CR25] Wang, F. H., Zevin, J. D., & Mintz, T. H. (2017). Top-down structure influences learning of nonadjacent dependencies in an artificial language. *Journal of Experimental Psychology: General,**146*(12), 1738. 29251987 10.1037/xge0000384

[CR26] Wang, F. H., Zevin, J. D., & Mintz, T. H. (2019). Successfully learning non-adjacent dependencies in a continuous artificial language stream. *Cognitive Psychology,**113*, Article 101223. 10.1016/j.cogpsych.2019.101223 31212192

[CR27] Weyers, I., Männel, C., & Mueller, J. L. (2022). Constraints on infants’ ability to extract non-adjacent dependencies from vowels and consonants. *Developmental Cognitive Neuroscience,**57*, Article 101149. 36084447 10.1016/j.dcn.2022.101149 PMC9465114

